# The expression of vascular endothelial growth factor C and its receptors in non-small cell lung cancer

**DOI:** 10.1054/bjoc.2001.1882

**Published:** 2001-07

**Authors:** T Kajita, Y Ohta, K Kimura, M Tamura, Y Tanaka, Y Tsunezuka, M Oda, T Sasaki, Go Watanabe

**Affiliations:** First Department of Surgery, Kanazawa University School of Medicine, Takara-machi 13-1, Kanazawa, 920-8641, Japan; Department of Experimental Therapeutics, Cancer Research Institute, Kanazawa University, Kanazawa, Japan

**Keywords:** VEGF-C, lung cancer, lymph node metastasis, VEGFR

## Abstract

Expression of vascular endothelial growth factor (VEGF)-C and that of its receptors were assessed in non-small cell lung cancer. Immunohistochemistry revealed positive VEGF-C expression in 38.7% (24/62) of the patients studied. A significant positive correlation was found between VEGF-C in cancer cells and VEGF receptor-3 (VEGFR-3) in vascular endothelial cells, but not between VEGF-C in cancer cells and VEGFR-2 in endothelial cells. In this cohort of lung cancer patients, VEGF-C expression was significantly associated with lymph node metastasis, lymphatic vessel invasion, and worse outcomes after the operation. Although the independent prognostic impact of VEGF-C and VEGFR-3 was not clear, VEGFR-2 expression in endothelial cells retained the independency as the prognostic indicator. In light of these findings, we conclude that VEGF-C plays an important role in lymphatic invasion/metastasis and tumour progression in non-small cell lung cancer. © 2001 Cancer Research Campaign http://www.bjcancer.com


British Journal of Cancer (2001) 85(2), 255–260
© 2001 Cancer Research Campaign
doi: 10.1054/ bjoc.2001.1882, available online at http://www.idealibrary.com on IDEAL®

http://www.bjcancer.com

### The expression of vascular endothelial growth factor C and its receptors in non-small cell lung cancer

T Kajita[1], Y Ohta[1], K Kimura[1], M Tamura[1], Y Tanaka[1], Y Tsunezuka[1], M Oda[1], T Sasaki[2] and Go Watanabe[1]

[1]First Department of Surgery, Kanazawa University School of Medicine, Takara-machi 13-1, Kanazawa 920-8641, Japan; [2]Department of Experimental Therapeutics, Cancer Research Institute, Kanazawa University, Kanazawa, Japan

**Summary** Expression of vascular endothelial growth factor (VEGF)-C and that of its receptors were assessed in non-small cell lung cancer. Immunohistochemistry revealed positive VEGF-C expression in 38.7% (24/62) of the patients studied. A significant positive correlation was found between VEGF-C in cancer cells and VEGF receptor-3 (VEGFR-3) in vascular endothelial cells, but not between VEGF-C in cancer cells and VEGFR-2 in endothelial cells. In this cohort of lung cancer patients, VEGF-C expression was significantly associated with lymph node metastasis, lymphatic vessel invasion, and worse outcomes after the operation. Although the independent prognostic impact of VEGF-C and VEGFR-3 was not clear, VEGFR-2 expression in endothelial cells retained the independency as the prognostic indicator. In light of these findings, we conclude that VEGF-C plays an important role in lymphatic invasion/metastasis and tumour progression in non-small cell lung cancer. © 2001 Cancer Research Campaign http://www.bjcancer.com

**Keywords**: VEGF-C; lung cancer; lymph node metastasis; VEGFR

Tumour progression is regulated by a number of stimulators and inhibitors of tumour angiogenesis (Folkman, 1995). Among the angiogenesis stimulators, vascular endothelial growth factor (VEGF) has been shown to be the most powerful mitogen for endothelial cells. Among VEGF members, VEGF-C has recently been found to induce not only angiogenesis but also lymphangiogenesis via VEGF receptor-2 (VEGFR-2) and VEGF receptor-3 (VEGFR-3) (Mustonen et al, 1995; Cao et al, 1998; Yonekura et al, 1999). A few researchers have reported the possible association of VEGF-C expression with intratumoural lymphatic vessel density or lymph node metastasis in some neoplasms (Ohta et al, 1999; Tsurusaki et al, 1999; Yonemura et al, 1999; Akagi et al, 2000; Niki et al, 2000). Of importance, some studies have recently shown that VEGF-C-mediated tumour lymphangiogenesis promotes cancer metastasis including nodal metastasis (Mandriota et al, 2001; Skobe et al, 2001). In the present study, we assessed the expression of VEGF-C and its receptors to better delineate their association with lymph node metastasis and cancer progression in non-small cell lung cancer.

### MATERIALS AND METHODS

### Tissue samples

Tumour samples were randomly obtained from 62 patients who had received surgery in the Kanazawa University Hospital between 1996 and 1997. For the immunohistochemical study, consecutive 6 μm-thick sections were cut from the tumour margin. The pathologic classification of each sample was confirmed by a review of haematoxylin and eosin (H&E)-stained sections. The pathological types were 36 adenocarcinomas, 20 squamous cell carcinomas, 2 adenosquamous carcinomas and 4 large cell carcinomas. The pathological stages were classified as stage I in 28 patients, II in 7, III in 26 and IV in 1 according to the Japanese Lung Cancer Society Classification.

*Received 24 October 2000*
*Revised 27 March 2001*
*Accepted 27 March 2001*

*Correspondence to:* Y Ohta

### Immunohistochemistry for VEGF-C and VEGFRs

The primary antibodies used in this study were an anti-VEGF-C goat polyclonal antibody at 1:200 dilution (Santa Cruz Biotechnology, Inc, Santa Cruz, CA, USA), an anti-VEGFR-3 rabbit polyclonal antibody at 1:200 dilution (Santa Cruz Biotechnology, Inc, Santa Cruz, CA, USA), an anti-VEGFR-2 rabbit monoclonal antibody at 1:200 dilution (Santa Cruz Biotechnology, Inc, Santa Cruz, CA, USA) and an anti-CD34 mouse monoclonal antibody (Nichirei, Tokyo, Japan). Paraffin sections were deparaffinized and immunohistochemical staining was performed using the immunoperoxidase technique. Endogenous peroxidase was blocked by treatment with 0.3% hydrogen peroxide in methanol for 15 min, and the sections were washed with Dulbecco’s phosphate-buffered saline (pH 7.2) without calcium ion or magnesium ion (PBS–). Tissue non-specific binding sites were blocked by 10% normal rabbit serum for VEGF-C, and by 10% normal goat serum for VEGFR-2, VEGFR-3 and CD34 for 15 min at room temperature. After they had been washed with PBS-, the sections were reacted with antibodies for 16 h at 4°C. Then they were washed with PBS-and reacted with biotinylated second antibody for 30 min at room temperature. After they had been washed with PBS-, avidin-biotin-peroxidase complex (DAKO LSAB2 system, DAKO Co, Carpinteria, CA, USA) was added and colour was developed using 3-3′-diaminobenzidine (Sigma, St Louis, MO, USA) with 0.03% hydrogen peroxide. Counterstaining was done with haematoxylin. For the negative control, all reagents except for the primary antibody were used. The specificities of VEGF-C and VEGFR-3 were tested using a blocking peptide (Santa Cruz Biotechnology, Inc, Santa Cruz, CA, USA).

### Assessment of microvessel density and immunohistochemical assessment of VEGF-C, VEGFR-2, and VEGFR-3

The total number of vascular endothelial cells was determined by immunohistochemistry for CD34 (Figure 1). The number was assessed blindly by 2 investigators. After the areas of highest vascularization had been chosen under low power (100 × magnification), vessel count within tumours was carried out in 3 fields on the screen image which was captured at 200 × magnification. The average counts of the 3 fields were recorded, and the mean value of the 2 investigators was used for the total number. For evaluation of immunostainings, cases in which at least 5% of tumour cells were immunoreactive were defined as being positive. As for the VEGFR-2 and VEGFR-3 staining within tumour cells, cases in which more than 50% of tumour cells were immunoreactive were defined as being strongly positive. As for the VEGFR-2 and VEGFR-3 staining within endothelial cells, after counting the total number of endothelial cells by CD34 staining as described above, the number of VEGFR-2- or VEGFR-3-positive endothelial cells was determined in the same manner by using consecutive paraffin sections. Cases in which at least 5% of endothelial cells were immunoreactive were defined as being positive in this study.

### Western blot analysis of VEGFR-3 in human lung cancer cell lines

5 human lung cancer cell lines (PC-3, PC-8, PC-10, PC-14, QG-56) were used and maintained in RPMI-1640 media (Gibco BRL, Gaithersburg, MD, USA) supplemented with 10% fetal bovine serum (FBS), 2 mM L-glutamine, 10 mM HEPES buffer, 500 units $ml^{-1}$ pencillin and 500 units $ml^{-1}$ streptomycin at 37°C and 5% $CO_2$. HUVEC was used as a positive control for VEGFR-3 and maintained in medium 199 (Life Technologies, Grand Island, NY, USA) supplemented with 20% FBS, 100 μg $ml^{-1}$ EC growth supplement, 50 U $ml^{-1}$ heparin, glutamine and antibiotics. 20 μg of protein sample were obtained from each cell line and mixed with 6 μl of sample buffer (50 mM Tris-HCL (pH 6.5), 10% glycerol, 2% SDS, and 0.1% bromphenol blue) prior to separation by 10% sodium dodecyl sulfate-polyacrylamide gel, followed by electrophotetic transfer of the proteins to a PVDF membrane (Millipore, Bedford, MA). The transferred samples were reacted with an anti-VEGFR-3 polyclonal antibody at 1:1000 dilution (Santa Cruz Biotechnology, Inc, Santa Cruz, CA, USA) and anti-β-actin monoclonal antibody (Sigma Chemical Co, St Louis, MO) for 2 h at room temperature. Secondary antibody, anti-mouse coupled to horseradish peroxidase, was incubated with the membrane and then washed in TBS-T. The presence of secondary antibody bound to membrane was detected by using the ECL Western blotting detection reagent (Amersham Life Science, Buckinghamshire, UK). As a negative control, primary VEGFR-3 antibody, which was pre-absorbed with the blocking peptide (Santa Cruz Biotechnology, Inc, Santa Cruz, CA, USA) overnight was used.

### Statistics

Fisher's exact test was used to examine the association between different variables and clinicopathological factors. Survival curves were obtained using the Kaplan–Meier method and compared using the log-rank test. A multivariate model using the Cox stepwise regression analysis was used to evaluate the statistical strength of independent association between selected covariates and patient survival. A *P* value less than 0.05 was considered significant.

### RESULTS

### VEGF-C, VEGFR-2 and VEGFR-3 expression in lung cancer tissue

VEGF-C antigen was observed in the cytoplasm of cancer cells (Figure 2). No staining was observed in the normal lung tissue. VEGF-C expression was positive in 38.7% (24/62) of the lung cancer patients. VEGFR-2 and VEGFR-3 antigens were observed in the cytoplasm of endothelial cells and cancer cells (Figure 3). The percentages of patients with positive VEGFRs in endothelial cells were 64.5% (40/62) for VEGFR-3 and 74.1% (46/62) for VEGFR-2. The percentages of patients with positive VEGFRs in cancer cells were 77.4% (48/62) for VEGFR-3 and 100% (62/62) for VEGFR-2. The percentages of the patients with strongly positive VEGFR staining in cancer cells were 27.4% (17/62) for VEGFR-3 and 51.6% (32/62) for VEGFR-2. A significant positive correlation was found between VEGF-C expression in cancer cells

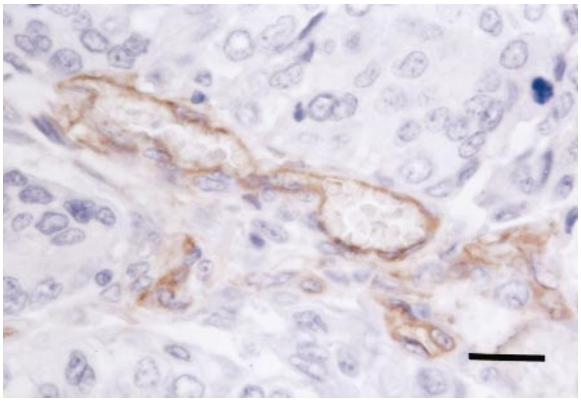

**Figure 1** Example of immunohistochemical staining for antibody against CD34. Scale bar, 25 μm

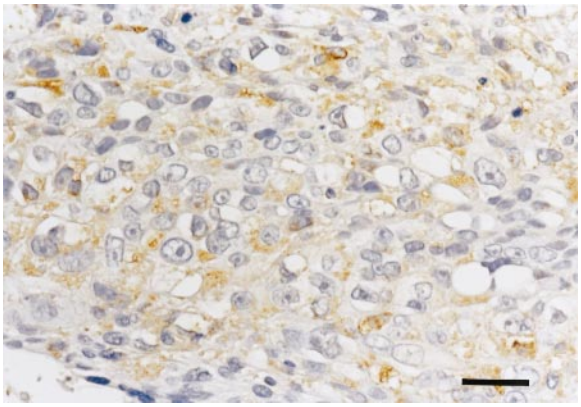

**Figure 2** Immunohistochemical staining for VEGF-C. Cytoplasmic staining was positive in tumour cells. Scale bar, 50 μm

© 2001 Cancer Research Campaign

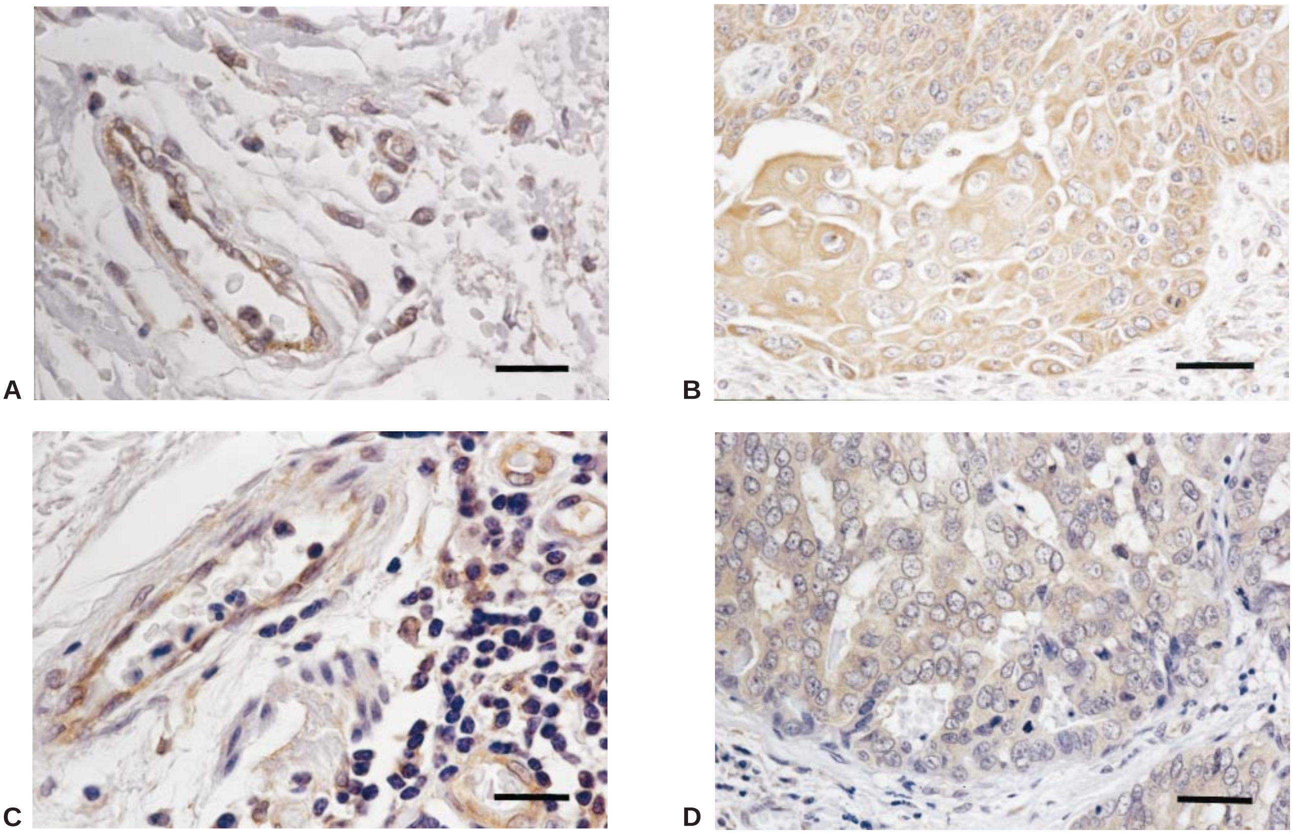

**Figure 3** Immunohistochemical staining for VEGFR-2 and VEGFR-3. Stainings of VEGFR-2 were identified in the vascular endothelial cells (**A**) (scale bar, 25 μm) and cytoplasm of tumour cells (**B**) (scale bar, 50 μm). VEGFR-3 staining was also found in the endothelial cells (**C**) (scale bar, 25 μm) and cytoplasm of tumour cells (**D**) (scale bar, 50 μm)

and VEGFR-3 expression in endothelial cells ($P < 0.01$), but not between VEGF-C in cancer cells and VEGFR-2 in endothelial cells (Table 1). Moreover, the expression of VEGF-C in cancer cells was also significantly associated with the intensities of both VEGFR-2 and VEGFR-3 staining in cancer cells (both $P < 0.05$) (Tables 1, 2). As a result of the Western blot analysis, a $M_r$ 125 000 band corresponding to VEGFR-3 protein was detected in all 5 lung cancer cell lines used (Figure 4).

### Correlations between VEGF-C expression and clinicopathological factors

Table 3 shows the correlations between clinicopathological factors and VEGF-C expression. VEGF-C expression was more frequently found in tumours with lymph node metastasis than in those without it ($P < 0.05$). VEGF-C expression in tumours with lymphatic invasion was significantly greater than that in tumours

**Table 1** Correlation between VEGF-C and VEGFR-3 expression

| Expression of VEGF-C | No. of cases | No. (%) of cases with strong positive VEGFR-3 (T) | *P* value | No. (%) of cases with positive VEGFR-3 (EC) | *P* value |
|---|---|---|---|---|---|
| Positive | 24 | 10 (41.6%) | 0.045 | 21 (87.5%) | 0.003 |
| Negative | 38 | 7 (18.4%) | | 19 (50.0%) | |

VEGFR-3 (T), VEGFR-3 expression in tumour cells; VEGFR-3 (EC), VEGFR-3 expression in endothelial cells.

**Table 2** Correlation between VEGF-C and VEGFR-2 expression

| Expression of VEGF-C | No. of cases | No. (%) of cases with strong positive VEGFR-2 (T) | *P* value | No. (%) of cases with positive VEGFR-2 (EC) | *P* value |
|---|---|---|---|---|---|
| Positive | 24 | 17 (70.8%) | 0.016 | 19 (79.1%) | 0.476 |
| Negative | 38 | 15 (39.4%) | | 27 (71.0%) | |

VEGFR-2 (T), VEGFR-2 expression in tumour cells; VEGFR-2 (EC), VEGFR-2 expression in endothelial cells.

© 2001 Cancer Research Campaign

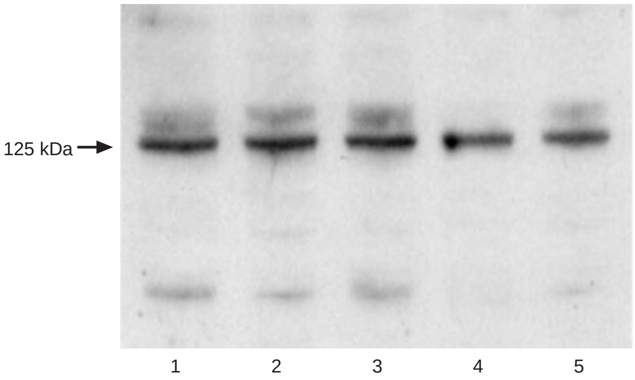

**Figure 4** Western blot analysis of VEGFR-3 expression in lung cancer cell lines. Lane 1, PC-3; Lane 2, PC-8; Lane 3, PC-10; Lane 4, PC-14; Lane 5, QG-56

without lymphatic invasion ($P < 0.05$). The VEGF-C expression was also significantly associated with gender and pathological stage (both $P < 0.05$) (Table 3).

### Correlations of clinicopathological and biological factors with survival

Figure 5 shows the survival of patients according to the VEGF-C status. Patients with VEGF-C expression had significantly poorer outcomes compared to those without ($P < 0.05$). From multivariate analysis, VEGFR-2 expression in endothelial cells, T factor and N factor retained independent negative prognostic impact on overall survival (Table 4).

### DISCUSSION

The findings of the current study demonstrated the positive association of VEGF-C expression with both lymph node metastasis and lymphatic invasion in lung cancer. In addition, a significant positive correlation was clearly found between VEGF-C expression in cancer cells and VEGFR-3 expression in endothelial cells. A positive association of VEGF-C expression with lymph node metastasis has been reported in gastric cancer, prostatic cancer, lung adenocarcinoma, and colorectal cancer (Tsurusaki et al, 1999; Yonemura et al, 1999; Akagi et al, 2000; Niki et al, 2000). A significant correlation between VEGF-C and VEGFR-3 has also been found in malignant mesothelioma and gastric cancer (Ohta et al, 1999; Yonemura et al, 1999). Our results indicated the possibility that VEGF-C produced in lung cancer cells modulates lymphatic metastasis through its receptor, VEGFR-3, in endothelial cells.

To our knowledge, this is the first report that described the prognostic aspect of VEGF-C expression in lung cancer. The lung cancer patients with VEGF-C expression had significantly poorer outcomes compared to those without it. Although we could find no prognostic independency in VEGF-C and VEGFR-3, the VEGFR-2 expression in endothelial cells retained significance. Since

**Table 3** Correlation between VEGF-C expression and clinicopathological factors

| Factors | No. of cases | Case no. (%) of VEGF-C positive | *P* value |
|---|---|---|---|
| Sex | | | |
| Male | 45 | 21 (46.6) | 0.036 |
| Female | 17 | 3 (17.6) | |
| Age | | | |
| ≤ 65 | 24 | 8 (33.3) | 0.489 |
| > 65 | 38 | 16 (42.1) | |
| Histology | | | |
| Adenocarcinoma | 36 | 9 (25.0) | 0.320 |
| Squamous cell carcinoma | 30 | 12 (60.0) | |
| Adenosquamous cell carcinoma | 2 | 2 (100.0) | |
| Large cell carcinoma | 4 | 1 (25.0) | |
| Histopathological grading | | | |
| Well differentiated | 21 | 6 (28.5) | 0.059 |
| Moderately differentiated | 21 | 8 (38.0) | |
| Poorly differentiated | 15 | 8 (53.3) | |
| T factor | | | |
| T1 | 22 | 7 (31.8) | 0.117 |
| T2, 3, 4 | 40 | 21 (52.5) | |
| N factor | | | |
| Positive | 25 | 14 (56.0) | 0.021 |
| Negative | 37 | 10 (27.0) | |
| Ly invasion | | | |
| Positive | 24 | 14 (58.3) | 0.011 |
| Negative | 38 | 10 (26.3) | |
| v invasion | | | |
| Positive | 20 | 9 (45.0) | 0.480 |
| Negative | 42 | 15 (35.7) | |
| Stage | | | |
| I, II | 25 | 9 (36.0) | 0.718 |
| III, IV | 37 | 15 (40.5) | |

© 2001 Cancer Research Campaign

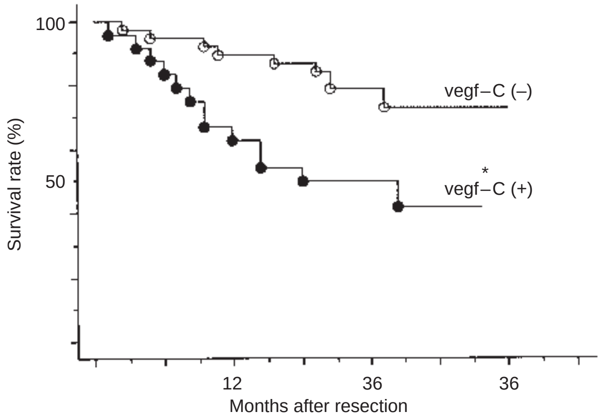

**Figure 5** Kaplan–Meier survival plots for lung cancers subdivided according to the VEGF-C expression. The difference in survival between the positive group (closed circle) and negative group (open circle) was significant (* $P < 0.05$)

**Table 4** Results of Cox hazard model analysis of prognostic factors (multivariate)

| Parameters | $\chi^2$ | *p* value | 95% CI |
|---|---|---|---|
| VEGF-C positive | 2.73 | 0.099 | 0.86–5.83 |
| VEGFR-2 (EC) positive | 8.54 | 0.004 | 0.10–0.64 |
| T1 vs. T2–4 | 6.82 | 0.009 | 1.71–42.06 |
| N0 vs. N1–3 | 9.07 | 0.003 | 2.33–54.60 |
| Stage I, II vs. III, IV | 2.58 | 0.108 | 0.05–1.34 |

VEGFR-2, which is a receptor for VEGF, VEGF-C, VEGF-D and VEGF-E, is known to also act as the main regulator of endothelial cell proliferation and migration (Waltenberger et al, 1994; Joukov et al, 1996; Ferrara and Davis-Smyth 1997; Achen et al, 1998; Meyer et al, 1999), we speculate that VEGFR-2 plays a key role in tumour angiogenesis and tumour progression/development in lung cancer.

It is notable that our immunohistochemical study showed positive VEGFR stainings not only in endothelial cells but also in cancer cells. Both VEGFR-1 and VEGFR-2 expressions have been observed in breast cancer, ovarian cancer, prostatic cancer and melanoma in addition to lung cancer (Gitay-Goren et al, 1993; Boocock et al, 1995; Lu and Brodie, 1996; Decaussin et al, 1999; Ferrer et al, 1999; Speirs and Atkin 1999; Xie et al, 1999). On the other hand, although the existence of VEGFR-3 expression in cancer cells has been reported in malignant mesothelioma, Kaposi's sarcoma, vascular tumour and melanoma (Pajusola et al, 1992; Jussila et al, 1998; Lymboussaki et al, 1998; Ohta et al, 1999), there has been no report on VEGFR-3 expression within lung cancer cells. Our results demonstrated here seem to strengthen the notion that lung cancer cells express VEGFR-3. Considering the positive association found between VEGF-C expression in lung cancer cells and VEGFR-2/VEGFR-3 stainings in cancer cells, the role of VEGF-C may encompass the tumour growth regulation in both paracrine and autocrine fashion. Although the prognostic impact of VEGFRs expression within cancer cells was not clear in this study, examination of the activation status through tyrosin phosphokinase is required to further elucidate the roles of VEGFRs in lung cancer cells.

In conclusion, this study enlightens the evidence that VEGF-C expression within cancer cells and VEGFR-3 expression within endothelial cells are of influence on the lymphatic metastasis in non-small cell lung cancer. Further analysis concerning lymphangiogeneis within tumour should be a promising way to understand the mechanism underlying the lymphatic metastasis in non-small cell lung cancer.

### REFERENCES

Achen MG, Jeltsch M, Kukk E, Makinen T, Vitali A, Wilks AF, Alitalo K and Stacker SA (1998) Vascular endothelial growth factor D (VEGF-D) is a ligand for the tyrosine kinases VEGF receptor-2 (Flk-1) and VEGF receptor-3 (Flt-4). *Proc Natl Acad Sci USA* **95**: 548–553

Akagi K, Ikeda Y, Miyazaki M, Abe T, Kinoshita J, Maehara Y and Sugimachi K (2000) Vascular endothelial growth factor-C (VEGF-C) expression in human colorectal cancer tissues. *Br J Cancer* **83**: 887–891

Boocock CA, Charnock-Jones DS, Sharkey AM, McLaren J, Barker PJ, Wright KA, Twentyman PR and Smith SK (1995) Expression of vascular endothelial growth factor and its receptors flt and KDR in ovarian carcinoma. *J Nat Cancer Inst* **87**: 506–516

Cao Y, Linden P, Farnebo J, Cao R, Eriksson A, Kumar V, Qi JH, Claesson WL and Alitalo K (1998) Vascular endothelial growth factor C induces angiogenesis in vivo. *Proc Natl Acad Sci USA* **95**: 14389–14394

Decaussin M, Sartelet H, Robert C, Moro D, Claraz C, Brambilla C and Brambilla E (1999) Expression of vascular endothelial growth factor (VEGF) and its two receptors (VEGF-R1-Flt-1 and VEGF-R2 Flk1/KDR) in non-small cell lung carcinomas (NSCLCs). *J Pathol* **188**: 369–377

Ferrara N and Davis-Smyth (1997) The biology of vascular endothelial growth factor. *Endocrine Rev* **18**: 4–25

Ferrer FA, Miller LJ, Lindquist R, Kowalczyk P, Laudone VP, Albertsen PC and Kreutzer DL (1999) Expression of vascular endothelial growth factor receptors in human prostete cancer. *Urology* **54**: 567–572

Folkman J (1995) Angiogenesis in cancer, vascular, rheumatoid and other disease. *Nature Med* **1**: 27–31

Gitay-Goren H, Halaban R and Neufeld G (1993) Human melanoma cells but not normal melanocytes express vascular endothelial growth factor receptors. *Biochem Biophys Res Commun* **190**: 702–708

Joukov V, Pajusola K, Kaipaien A, Chilov D, Lahtinen I, Kukk E, Saksela O, Kalkkinen N and Alitalo K (1996) A novel vascular endothelial growth factor, VEGF-C, is a ligand for the Flt-4 (VEGFR-3) and KDR (VEGFR-2) receptor tyrosine kinases. *EMBO J* **15**: 290–298

Jussila L, Valtola R, Partanen TA, Salven P, Heikkila P, Matikainen M-T, Renkonen R, Kaipainen A, Detmar M, Tschachler E, Alitalo R and Alitalo K (1998) Lymphatic endothelium and Kaposi's sarcoma spindle cells detected by antibodies against the vascular endothelial growth factor receptor-3. *Cancer Res* **58**: 1599–1604

Lu Q and Brodie A (1996) Stimulation of the growth of MCF-7 and MDA MB-468 breast cancer cells by vaslular endothelial growth factor. *Proc Am Assoc Cancer Res* **37**: 1499

Lymboussaki A, Partanen TA, Olofsson B, Thomas-Crusells J, Fletcher C, deWaal R, Kaipainen A and Alitalo K (1998) Expression of the vascular endothelial growth factor C receptor VEGFR-3 in lymphatic endothelium of the skin and in vascular tumors. *Am J Pathol* **153**: 395–403

Mandriota SJ, Jussila L, Jeltsch M, Compagni A, Baetens D, Prevo R, Banerji S, Huarte J, Montesano R, Jackson DG, Orci L, Alitalo K, Christofori G and Pepper MS (2001) Vascular endothelial growth factor-C-mediated lymphangiogenesis promotes tumour metastasis. *EMBO J* **20**: 672–682

Meyer M, Clauss M, Lepple-Wienhues A, Waltenberger J, Augustin HG, Ziche M, Lanz C, Buttner M, Rziha HJ and Dehio C (1999) A novel vascular endothelial growth factor encoded by Orf virus, VEGF-E, mediates angiogenesis via signalling through VEGFR-2 (KDR) but not VEGFR-1 (Flt-1) receptor tyrosine kinases. *EMBO J* **18**: 363–374

Niki T, Iba S, Tokunou M, Yamada T, Matsuno Y and Hirohashi S (2000) Expression of vascular endothelial growth factors A, B, C, and D and their relationships to lymph node status in lung adenocarcinoma. *Clin Cancer Res* **6**: 2431–2439

Ohta Y, Shridhar V, Bright RK, Kalemkerian GP, Du W, Carbone M, Watanabe Y and Pass HI (1999) VEGF and VEGF type C play an important role in angiogenesis and lymphangiogenesis in human malignant mesothelioma tumours. *Br J Cancer* **81**: 54–61

*© 2001 Cancer Research Campaign*

Pajusola K, Aprelikova O, Korhonen J, Kaipainen A, Pertovaara L, Alitalo R and Alitalo K (1992) FLT-4 receptor tyrosine kinase contains seven immunoglobulin-like loops and is expressed in multiple human tissues and cell lines. *Cancer Res* **52**: 5738–5743

Skobe M, Hawighorst T, Jackson D, Prevo R, Janes L, Velasco P, Riccardi L, Alitalo K, Claffey K and Detmar M (2001) Induction of tumor lymphangiogenesis by VEGF-C promotes breast cancer metastasis. *Nature med* **7**: 192–198

Speirs V and Atkin SL (1999) Production of VEGF and expression of the VEGF receptors Flt-1 and KDR in primary cultures of epithelial and stromal cells derived from breast tumours. *Br J Cancer* **80**: 898–903

Tsurusaki T, Kanda S, Sakai H, Kanetake H, Saito Y, Alitalo K and Koji T (1999) Vascular endothelial growth factor-C expression in human prostatic carcinoma and its relationship to lymph node metastasis. *Br J Cancer* **80**: 309–313

Waltenberger J, Claesson-Welsh L, Siegbahn A, Shibuya M and Heldin CH (1994) Different signal transduction properties of KDR and Flt-1, two receptors for vascular endothelial growth factor. *J Biol Chem* **269**: 26988–26995

Xie B, Tam NNC, Tsao SW and Wong YC (1999) Co-expression of vascular endothelial growth factor (VEGF) and its receptors (flk-1 and flt-1) in hormone induced mammary cancer in the Noble rat. *Br J Cancer* **81**: 1335–1343

Yonekura H, Sakurai S, Liu X, Migita H, Wang H, Yamagishi S, Nomura M, Abedin MJ, Unoki H, Yamamoto Y and Yamamoto H (1999) Placenta growth factor and vascular endothelial growth factor B and C expression in microvascular endothelial cells and pericytes. Implication in autocrine and paracrine regulation of angiogenesis. *J Biol Chem* **274**: 35172–35178

Yonemura Y, Endo Y, Fujita H, Fushida S, Ninomiya I, Bandou E, Taniguchi K, Miwa K, Ohoyama S, Sugiyama K and Sasaki T (1999) Role of vascular endothelial growth factor C expression in the development of lymph node metastasis in gastric cancer. *Clin Cancer Res* **5**: 1823–1829

*© 2001 Cancer Research Campaign*
